# Childhood trauma, peer victimization, and non-suicidal self-injury among Chinese adolescents: a latent variable mediation analysis

**DOI:** 10.1186/s12888-023-04848-z

**Published:** 2023-06-15

**Authors:** Ke Zhao, Siyu Tong, Lan Hong, Shang Yang, Wenyun Yang, Yao Xu, Zilin Fan, Jiaqi Zheng, Keqing Yao, Tiansheng Zheng

**Affiliations:** 1 Lishui Second People’s Hospital Afliated to Wenzhou Medical University, Lishui, 323000 China; 2 School of Mental Health, Wenzhou Medical University, Wenzhou, 325000 China; 3 The Affiliated Kangning Hospital of Wenzhou Medical University Zhejiang Provincial Clinical Research Center for Mental Disorder, Wenzhou, 325000 China; 4 The Third Hospital of Quzhou, Quzhou, 324000 China; 5 Shenzhen Mental Health Center, Shenzhen, Guangdong China; 6 Shenzhen Kangning Hospital, 77 Zhenbi Road, Pingshan District, Shenzhen, Guangdong 518118 China

**Keywords:** Non-suicidal self-injury, Childhood trauma, Peer victimization, Chinese adolescents, Latent variable, Mediation analysis

## Abstract

**Background:**

Childhood and peer experiences can influence adolescents’ perceptions of interpersonal relationships, which can, in turn, influence their emotional states and behavior patterns. Non-suicidal self-injury (NSSI) is now a common problem behavior among adolescents. The present study examined the role of childhood trauma and peer victimization in adolescents’ NSSI.

**Methods:**

A cross-sectional survey was conducted among 1783 adolescents (1464 girls and 318 boys) in the psychiatric outpatient clinics or wards of 14 psychiatric hospitals or general hospitals in nine provinces in China. Data were collected using the Multidimensional Peer Victimization Scale (MPVS), Short-form Childhood Trauma Questionnaire(CTQ-SF), and Functional Assessment of Self-Mutilation (FASM). Structural equation modeling (SEM) with latent variables was used to demonstrate the mediating role of peer victimization in the association between childhoodtrauma and NSSI.

**Results:**

The SEM analysis demonstrated that peer victimization plays a partial mediating role in the relationship between childhood trauma and NSSI. In addition, several covariates (such as age, gender, education level, and place of residence) effectively regulated the relationship between peer victimization and NSSI.

**Conclusion:**

In future studies of NSSI among Chinese adolescents, attention should be paid to the roles of childhood trauma and peer bullying; there is a temporal sequence between these two variables and, to some extent, childhood trauma can have an impact on bullying during adolescence which, in turn, influences NSSI behavior.

**Supplementary Information:**

The online version contains supplementary material available at 10.1186/s12888-023-04848-z.

## Introduction

Non-suicidal self-injury (NSSI) is defined as the deliberate direct destruction or alteration of body tissue without conscious suicidal intent [1]. In the Diagnostic and Statistical Manual of Mental Disorders, fifth edition (DSM-5), this diagnosis is denoted as a “ condition for further study” [2]. Globally, the lifetime prevalence of at least one episode of NSSI in adolescence is 17–18% [3]. As early as 2016, the prevalence of adolescent NSSI was found to be 15.0% in the sample of Chinese adolescent communities [4]. Besides, a large-scale sampling survey in 2021 showed that the prevalence of NSSI was 28.5% among 18,900 Chinese junior and senior high school students [5]. Besides, a recent study reported that 33.7–51% of community youths engage in self-injurious behavior [6]. Moreover, a meta-analysis showed that individuals with mood disorders (anxiety, depression, bipolar, and related disorders) are at higher risk of NSSI compared to normal controls [7]. In an adolescent psychiatric sample, the prevalence of one-time-only NSSI behavior was as high as 60%, and the incidence of recurrent NSSI was approximately 50% [8]. A better understanding of the underlying mechanisms and risk factors for adolescent NSSI is crucial to inform prevention and intervention efforts.

Childhood trauma includes abuse and neglect by family members, especially primary caregivers [9], and has been identified as an important risk factor for the occurrence of NSSI [10] [11]. Neglect can be divided into two categories [12]: (1) physical neglect, where the basic requirements for the child’s survival are not met, such as nutrition and shelter [13].; (2) emotional neglect, where the basic psychological needs of the child are neglected or diluted. Abuse can be divided into the following categories: (1) physical abuse, which is intentional and violent physical injury; (2) emotional abuse, which refers to verbal and other mental attacks that harm a child’s health, including attacks on self-confidence, insults, and other behaviors that hinder a child’s normal growth; (3) sexual abuse, defined as an adult using violence, inducement, or other methods to engage in any form of sexual behavior with a child [14].

The interdependent parent-child relationship is a unique feature of Chinese society and family relationships play an irreplaceable role in the lives of Chinese children [15]. According to attachment theory, children function best when in a safe environment created by their parents. However, if a child does not feel safe, a child’s mind attempts to reduce the threats and create safety by fighting, fleeing, or freezing [16]. When adolescents are exposed to danger, decisions are made instantaneously by the brain; this process primarily involves conditioned responses formed during early childhood rather than complex processes performed by higher-order parts of the brain that are not fully formed until early adulthood (i.e., the prefrontal and anterior cingulate cortex, the parts of the brain responsible for language, associative, and regulatory functions) [17]. That is, the early environment also affects adolescents’ coping styles and behaviors when facing risks [18]. To sum up, childhood experiences also have a fundamental role in subsequent development that cannot be ignored [15]. Importantly, a recent meta-analysis found that adverse childhood experiences have significant independent effects on self-harm [19]. Further, several studies have indicated that childhood trauma can directly [20] or indirectly lead to the development of NSSI [21]. Although there is a time lag, the association between childhood trauma and NSSI in adolescence is significant.

Peer victimization is when an individual is repeatedly and chronically bullied or victimized by one or more peers [22], including: (1) physical victimization, involving physical attacks by peers; (2) verbal victimization, involving verbal abuse by peers; (3) social victimization, involving bullying by peers in a personal relationship; and (4) property victimization, involving stealing or destruction of property by peers [23]. Individuals who have experienced peer victimization have a greater risk of NSSI [24]. In the highly developed modern society, the psychological growth of adolescents is inseparable from the establishment of social relationships with family members, peers, and teachers; peer relationships account for a large proportion of the life of adolescents [25]. The influence of peer relationships on adolescents should not be ignored. According to interpersonal suicide theory (ITS), the desire to die may increase if a sense of burden and frustrated belonging is perceived in core interpersonal interactions, and this desire then leads to self-injury and suicidal behavior Furthermore, integrative motivation-volition theory (IMV) suggests that negative life events, especially feelings that can be internalized as shame or frustration in interpersonal relationships, can lead to feelings of entrapment and subsequent suicidal thoughts and plans [26]. In a study of depressed adolescents, Vergara GA et al. reported that severe peer victimization and bullying led to NSSI [27]. Similarly, in another study, negative peer experiences were found to predict NSSI trajectories in depressed adolescents [28]. In summary, there is considerable evidence for a causal relationship between peer victimization and NSSI [29] [30].

In China, the saying goes ‘a single silk does not make a thread; a single tree does not make a forest’. Collectivism has always been a prominent characteristic of Chinese culture [31]. Even in modern society, there is a continuing cultural association between collectivism and some life domains [31]. In other words, Chinese people are more connected to the people around them, whether it is their family or friends. Several studies have reported that Chinese people have a higher level of dependency compared to people in Western countries [32]. Therefore, relationships between people are of extraordinary significance to the Chinese. Moreover, childhood trauma and peer victimization can be considered to be two different types of interpersonal trauma in adolescent life [33], both of which involve emotional and physical abuse [34]. Therefore, we hypothesized that childhood trauma and peer victimization may play significant roles in adolescent emotional and behavioral problems.

Although the correlation between childhood trauma and NSSI has been well confirmed in previous studies, to date, few studies have examined the mediating factors and underlying mechanisms of this relationship [35]. One of the existing studies found that adolescents who experienced more childhood abuse were also more likely to report peer victimization. In this prospective study, females who suffered childhood trauma (child sexual abuse) were at increased risk of peer victimization. Still, there is a need to explore in more depth the intrinsic links between childhood trauma, peer victimization, and NSSI. Further, at present, the majority of studies on family relationships, friendships, and NSSI are based on Western samples; these relationships have rarely been explored in the Chinese context. Due to the differences in the construction of society and the influence of traditional culture, there may be important differences between Chinese adolescents’ perceptions of interpersonal relationships and the importance they place on them, as compared to their Western counterparts.

In summary, although several risk factors for NSSI have been identified previously [36], there are few published studies on the effects of the external environment on adolescent NSSI. Further, most studies have measured childhood trauma and peer victimization as observable outcomes [37]; however, there are two types of variables: latent variables and observed variables. Latent variables are composed of a series of factor variables that constitute the latent variable, and if latent variables are treated as observed variables and processed directly, biased results may be obtained [38]. Thus, the mediation effect of peer victimization on the relationship between childhood trauma and NSSI was explored using (structural equation modelling) SEM in the current study to explore the influence of early family environment and peer relationships on the NSSI of Chinese adolescents.

## Methods

### Participants

The current study used data from the Chinese Adolescent Depression Cohort (CADC). Between December 2020 and December 2021, a total of 1783 adolescents (1464 girls and 318 boys) were recruited from the psychiatric outpatient clinics or wards of 14 psychiatric hospitals or general hospitals in nine provinces in China.

**Fig. 1:**
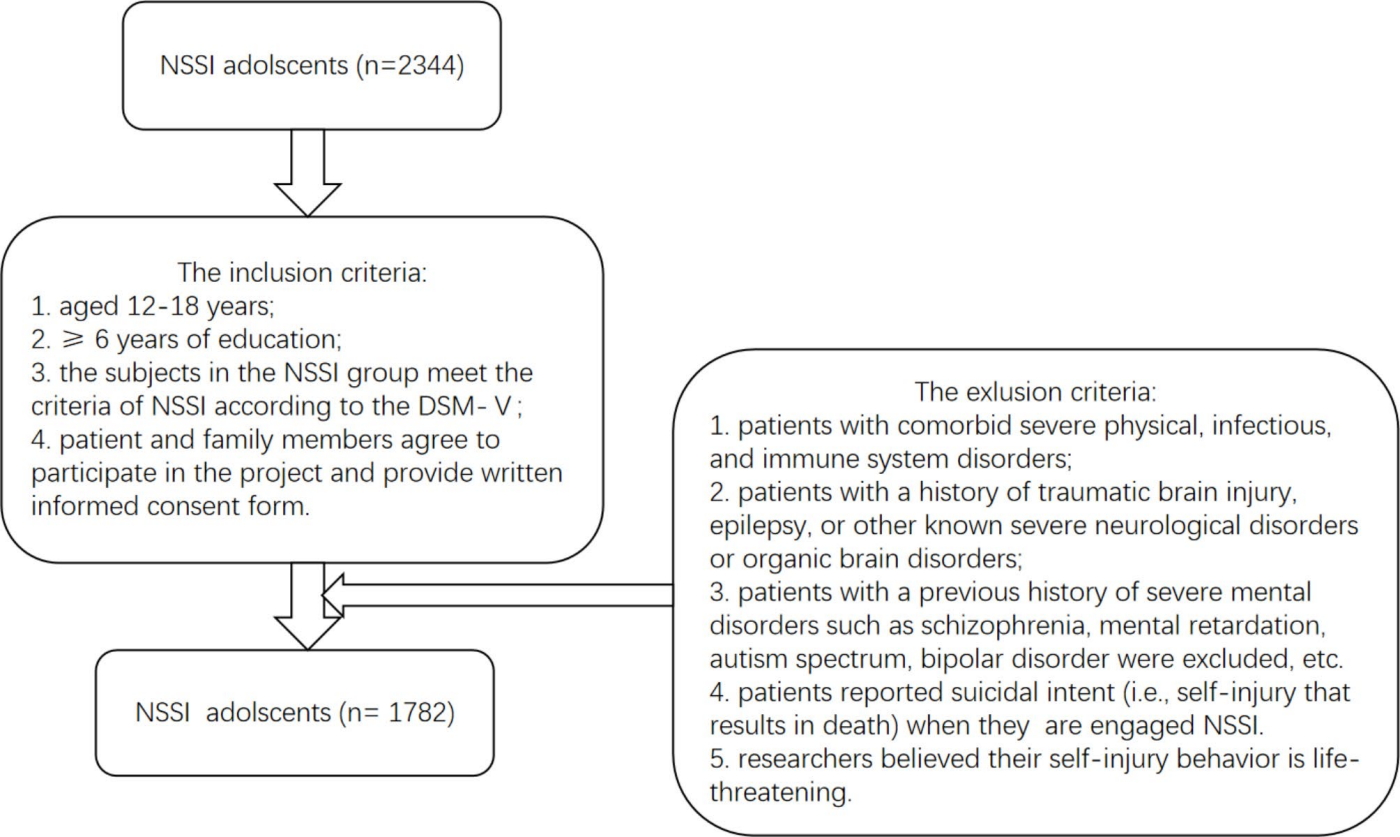
Sample flow chart. Note: NSSI Non-suicidal self-injury

The inclusion criteria included: (1)aged 12–18 years; (2) ≥ 6 years of education; (3) the subjects in the NSSI group meet the criteria of NSSI according to the DSM-V, see Additional file 1; (4) patient and family members agree to participate in the project and provide written informed consent form;

The exclusion criteria included: (1) patients with comorbid severe physical, infectious, and immune system disorders; (2) patients with a history of traumatic brain injury, epilepsy, or other known severe neurological disorders or organic brain disorders; (3) patients with a previous history of severe mental disorders such as schizophrenia, mental retardation, autism spectrum, bipolar disorder were excluded, etc. (Fig. 1). (4) patients reported suicidal intent (i.e., self-injury that results in death) when they are engaged NSSI [39]. (5) researchers believed their self-injury behavior is life-threatening.

### Procedure

All participants were evaluated by the psychiatric directors. The time of evaluation is about 15 min (some evaluation may take more time than other, which depend on the psychological status of the participants). After pass the evaluation, participants took the questionnaire test under the guidance of psychology or psychiatry graduate students. Prior to the official start of the study, all the researchers were trained to ensure they were familiar with the evaluation process. All tests are carried out in a quiet ward using a tablet computer and take approximately 30 min. The researchers introduce the project to the group of participants and legal guardians at the start of the study. Participants were asked to answer the questions truthfully. After completing the survey, the researcher presented the results to the participant and interpreted them accordingly. All participants provided written informed consent before taking part in the study. The study was approved by the Institutional Review Board (IRB) of Shenzhen Kangning Hospital, Ethics Approval No.: 2020-k021-02.

### Measures

#### Functional assessment of self-mutilation (FASM)

The FASM is a widely used tool to assess NSSI among adolescents. It is divided into 11 types of NSSI behavior and 22 functional domains of NSSI components [40]. The FASM was developed by Lloyd et al.[41]. In the current study, the Chinese version of the FASM was used [42], as this is more suitable for Chinese youth. The Cronbach’s α for the FASM in this study was 0.65.

#### Short-form childhood trauma questionnaire (CTQ-SF)

The CTQ scale was developed by Bernstein and Fink to detect experiences of child abuse and neglect [34]. It is divided into five subscales: emotional abuse, physical abuse, sexual abuse, emotional neglect, and physical neglect. There are 28 questions in total, and each question is scored on a five-point scale, with the total possible score ranging from 28 to 140; higher scores indicate higher levels of trauma exposure [12]. The scale has good reliability; the Cronbach’s α for the CTQ in this study was 0.84. Moreover, the α coefficients for the five sub-scales ranged from 0.70 (physical neglect) to 0.93(sexual abuse), indicating that the scale has good internal consistency.

#### Multidimensional peer victimization scale (MPVS)

The MPVS was developed by Mynard and Joseph. It is a 16-item self-report scale covering four dimensions of peer victimization: physical victimization, verbal victimization, social manipulation, and attacks on property [43]. Scores on each of the four dimensions range from 0 to 16, with higher scores indicating more incidents of peer victimization. The MPVS has good reliability and is suitable for measuring bullying victimization (internal consistency: Physical victimization = 0.85; Verbal victimization = 0.75; Social manipulation = 0.77; Property attacks = 0.73). The Cronbach’s α for the total MPVS score in this study was 0.89.

### Analytic procedures

The descriptive statistics were compared between the two groups using Welch’s t-tests and Fisher’s exact tests. Preliminary data analyses were conducted using SPSS 22.0 (IBM, Armonk, NY, USA).

Latent variable structural equation modelling (SEM) was adopted to analyze the mediation effect of multidimensional peer victimization on the relationship between childhood trauma and adolescent NSSI. SEM is a data analysis tool that combines path analysis [44] and the common factor model [45]. It can verify a conceptual model and a study’s research hypotheses by specifying relations among observed entities and hypothesized latent constructs [46]. Latent variable models allow researchers to address research questions that directly compare the viability of dimensional, categorical, and hybrid conceptions of constructs and provide greater validity and generalizability by allowing for the correction of measurement error, as compared to methods based on observed variables [47]. In this model, childhood trauma and peer victimization were considered “latent” variables with five observed variables (emotional abuse, physical abuse, sexual abuse, emotional neglect, and physical neglect) [34] and four observed variables (physical victimization, verbal victimization, social manipulation, and attacks on property) [23], respectively. NSSI was estimated as an “observed” variable. A bootstrapping procedure was used to test the direct and indirect effects of the model. The demographic variables age, gender, education, and residence were used as covariates in this study. All modelling procedures were performed using Mplus 8.4.

The fit of the model was tested using chi-square statistic (CMIN), degrees of freedom (df), comparative fit index (CFI), adjusted goodness-of-fit index (AGFI), goodness-of-fit index (GFI), root mean square error of approximation (RMSEA), and Akaike’s Information Criterion [48].

## Results

### Descriptive statistics

As can be seen in Table 1, the study cohort comprised 318 males (17.8%) and 1464 females (82.2%), ranging in age from 12 to 18 years (mean ± SD = 14.85 ± 1.82). Overall, 66.4% of participants were from urban areas and 33.6% were from rural areas. Approximately half of the participants were in junior high school (55.8%), 4.3% were in primary school, and 39.9% were in senior high school or above. Most of the participants’ parents had a middle school education or above (father, 86.9%; mother, 77.1%).

**Table 1:** Sociodemographic characteristics of participants (N = 1782)

Variable	n	%	M	SD	significant effect (p < 0.05)
Age			14.85	1.82	c, p, n
Gender					n
Male	318	17.8			
Female	1464	82.2			
Resident					c, p
Urban	1184	66.4			
Rural	598	33.6			
Education			9.05	1.75	c, p, n
Primary school	77	4.3			
Junior high school	994	55.8			
Senior high school or above	711	39.9			
CTQ			56.02	13.32	
MPVS			36.32	17.21	
NSSI			23.10	9.12	

**Note:** gender( male=”1”, female=”2”) and residence (urban=”1”, rural=”2”); Independent t-test and ANOVA were used to calculate the differences of CTQ, MPVS and NSSI in various demographic variables. ( c = CTQ; p = MPVS; n = NSSI);CTQ, Childhood Trauma Questionnaire; MPVS, Multidimensional peer victimization Scale; NSSI, non-suicide self-injury

The mean total CTQ, MPVS, and NSSI scores were 56.02 (± 13.32), 36.32(± 17.21), and 23.10 (± 9.12), respectively. Further, there were significant differences in NSSI, CTQ, and MPVS as a function of age, gender, residence, and education. Specifically, there were significant gender differences in NSSI, with women experiencing more NSSI behaviors than men; the significant differences in residence on PVQ and CTQ, with rural adolescents experiencing more peer bullying and childhood trauma than urban ones. Besides, there were significant Education level and age differences in NSSI, CTQ, MPVS and significant parental education differences in CTQ and MPVS.

### Correlation analysis

This study found that there was a significant positive correlation between NSSI and CTQ, MPVS; The age of demographic variables was negatively correlated with CTQ and MPVS; There is a significant positive correlation between gender and NSSI, but there is no significant correlation with total score of CTQ and MPVS, and there is a significant correlation with some factors of these two variables (CTQ, emotional abuse; MPVS, physical victimization, verbal victimization). Residence was not significantly associated with NSSI, but showed a significant association with CTQ, MPVS total score, and was not significantly associated with some of the factors of CTQ ( emotional abuse, sexual abuse, emotional neglect, physical neglect). Among demographic variables, age was negatively correlated with gender, and positively correlated with education level; There is a significant negative correlation between gender and education level; Other correlations were not significant(Table 2).

**Table 2:** Correlations between variables

	NSSI	CTQ	C1	C2	C3	C4	C5	PVQ	P1	P2	P3	P4	Age	Edu	Gen	Rsd
NSSI	1															
CTQ	**0.31** ^**^	**1**														
C1	**0.18** ^**^	**0.73** ^**^	1													
C2	**0.23** ^**^	**0.67** ^**^	**0.26** ^**^	1												
C3	**0.18** ^**^	**0.69** ^**^	**0.28** ^**^	**0.51** ^**^	1											
C4	**0.32** ^**^	**0.81** ^**^	**0.54** ^**^	**0.39** ^**^	**0.45** ^**^	1										
C5	**0.04** ^**^	**0.15** ^**^	**0.11** ^**^	-0.02	**-0.29** ^**^	0.03	1									
MPVS	**0.33** ^**^	**0.44** ^**^	**0.29** ^**^	**0.34** ^**^	**0.23** ^**^	**0.40** ^**^	**0.10** ^**^	1								
P1	**0.28** ^**^	**0.37** ^**^	**0.27** ^**^	**0.27** ^**^	**0.18** ^**^	**0.32** ^**^	**0.11** ^**^	**0.76** ^**^	1							
P2	**0.28** ^**^	**0.33** ^**^	**0.19** ^**^	**0.27** ^**^	**0.18** ^**^	**0.32** ^**^	**0.07** ^*^	**0.83** ^**^	**0.47** ^**^	1						
P3	**0.25** ^**^	**0.36** ^**^	**0.25** ^**^	**0.28** ^**^	**0.18** ^**^	**0.35** ^**^	**0.07** ^*^	**0.84** ^**^	**0.50** ^**^	**0.62** ^**^	1					
P4	**0.28** ^**^	**0.36** ^**^	**0.25** ^**^	**0.30** ^**^	**0.20** ^**^	**0.31** ^**^	**0.10** ^**^	**0.82** ^**^	**0.55** ^**^	**0.56** ^**^	**0.58** ^**^	1				
Age	**-0.17** ^**^	**-0.11** ^**^	-0.02	**-0.15** ^**^	**-0.12** ^**^	**-0.10** ^**^	**0.05** ^*^	**-0.25** ^**^	**-0.24** ^**^	**-0.17** ^**^	**-0.24** ^**^	**-0.18** ^**^	1			
Edu	**-0.16** ^**^	**-0.12** ^**^	-0.02	**-0.16** ^**^	**-0.12** ^**^	**− 0.10** ^**^	0.03	**-0.25** ^**^	**-0.25** ^**^	**-0.16** ^**^	**-0.23** ^**^	**-0.20** ^**^	**0.85** ^**^	1		
Gen	**0.09** ^**^	-0.02	**-0.10** ^**^	-0.02	0.02	0.04	0.02	-0.01	**-0.07** ^**^	**0.06** ^*^	-0.02	-0.03	**-0.12** ^**^	**-0.1** ^**^	1	
Rsd	0.03	**0.06** ^**^	-0.04	**0.17** ^**^	0.04	0.02	0.03	**0.12** ^**^	**0.07** ^**^	**0.07** ^**^	**0.11** ^**^	**0.14** ^**^	0.01	-0.01	-0.05	1

Note: * *p*<0.05, ** *p*<0.01; *P*<0.05 are marked boldface. gender ( male=”1”, female=”2”) and residence (urban=”1”, rural=”2”)

CTQ, Childhood Trauma Questionnaire; c1, emotional abuse; c2, physical abuse; c3, sexual abuse; c4, emotional neglect; c5, physical neglect; MPVS, Multidimensional peer victimization Scale; p1, physical victimization; p2, verbal victimization; p3, social manipulation; p4, attacks on property; Age, age; Gen, gender; Edu, education; Rsd, resident; Fedu, father’s education; Medu, mother’s education

### Regression analysis

On the various dimensions of CTQ, emotional abuse, physical abuse, emotional neglect, physical neglect have a significant impact on MPVS, and physical abuse and emotional neglect have a significant impact on NSSI; On the various dimensions of MPVS, physical victimization, verbal victimization and attacks on property have a significant impact on NSSI. In covariates, age, gender, education, resident had a significant impact on MPVS, while gender had no significant impact on MPVS; age and gender had a significant impact on NSSI, while the other covariates had no significant impact on NSSI; Those variables does not exist the serious multicollinearity. The results are shown in Tables 3 and 4.

**Table 3:** Linear Regression Analysis between CTQ, MPVS, Age, Gender, Education, Resident and NSSI.

Model		Unstandardized Coefficients		Standard coefficient	t	p	VIF
		B	SE	β			
Independent variable	(constants)	18.166	2.691		6.751	0.00	
	CTQ						
	C1	-0.017	0.053	-0.009	-0.331	0.740	1.506
	**C2**	**0.183**	**0.073**	**0.068**	**2.506** ^*^	**0.012**	**1.556**
	C3	-0.008	0.053	-0.004	-0.154	0.878	1.738
	**C4**	**0.391**	**0.055**	**0.206**	7.102**	**0.000**	**1.794**
	C5	0.04	0.077	0.012	0.513	0.608	1.191
	MPVS						
	**P1**	**0.36**	**0.098**	**0.104**	3.688**	**0.000**	**1.684**
	**P2**	**0.238**	**0.084**	**0.084**	2.823**	**0.005**	**1.885**
	P3	-0.01	0.093	-0.003	-0.103	0.918	2.015
	**P4**	**0.271**	**0.099**	**0.082**	2.744**	**0.006**	**1.904**
Control variables	**Age**	**-0.489**	**0.231**	**-0.088**	-2.113*	**0.035**	**3.709**
	**Gen**	**1.779**	**0.531**	**0.075**	3.348**	**0.001**	**1.062**
	Edu	0.055	0.216	0.011	0.253	0.801	3.69
	Rsd	-0.259	0.432	-0.013	-0.601	0.548	1.066
R2				0.173			
F				28.368			
P				<0.001			
Dependent variable: NSSI							

Note: * *p*<0.05, ** *p*<0.01; *P*<0.05 are marked boldface; VIF, variance inflation factor (VIF<10, those variables does not exist the serious multicollinearity) ;

CTQ, Childhood Trauma Questionnaire; c1, emotional abuse; c2, physical abuse; c3, sexual abuse; c4, emotional neglect; c5, physical neglect; MPVS, Multidimensional peer victimization Scale; p1, physical victimization; p2, verbal victimization; p3, social manipulation; p4, attacks on property; Age, age; Gen, gender; Edu, education; Rsd, resident

**Table 4:** Linear Regression Analysis between CTQ, Age, Gender, Education, Resident and MPVS.

Model		Unstandardized Coefficients		Standard Coefficie-nt	t	p	VIF
		B	SE	β			
	(constant)	12.421	2.587		4.801	0.000	
Independent variable	CTQ						
	**C1**	**0.192**	**0.051**	**0.093**	**3.761** ^**^	**0.000**	**1.484**
	**C2**	**0.478**	**0.07**	**0.170**	**6.793** ^**^	**0.000**	**1.515**
	C3	0.001	0.052	0.000	0.011	0.992	1.735
	**C4**	**0.505**	**0.052**	**0.256**	9.649**	**0.000**	**1.699**
	**C5**	**0.334**	**0.075**	**0.099**	4.476**	**0.000**	**1.172**
Control variables	**Age**	**-0.803**	**0.225**	**-0.140**	-3.575**	**0.000**	**3.67**
	Gen	-0.901	0.513	-0.037	-1.758	0.079	1.039
	**Education**	**-0.454**	**0.21**	**-0.084**	-2.162*	**0.031**	**3.659**
	**Resident**	**1.628**	**0.417**	**0.081**	3.901**	**0.000**	**1.048**
R2				0.265			
F				70.962			
P				0.000			
Dependent variable: MPVS							

Note: * *p*<0.05, ** *p*<0.01; *P*<0.05 are marked boldface. gender ( male=”1”, female=”2”) and residence (urban=”1”, rural=”2”)

CTQ, Childhood Trauma Questionnaire; c1, emotional abuse; c2, physical abuse; c3, sexual abuse; c4, emotional neglect; c5, physical neglect; MPVS, Multidimensional peer victimization Scale; p1, physical victimization; p2, verbal victimization; p3, social manipulation; p4, attacks on property; Age, age; Gen, gender; Edu, education; Rsd, resident

### Mediation analysis

**Fig. 2:**
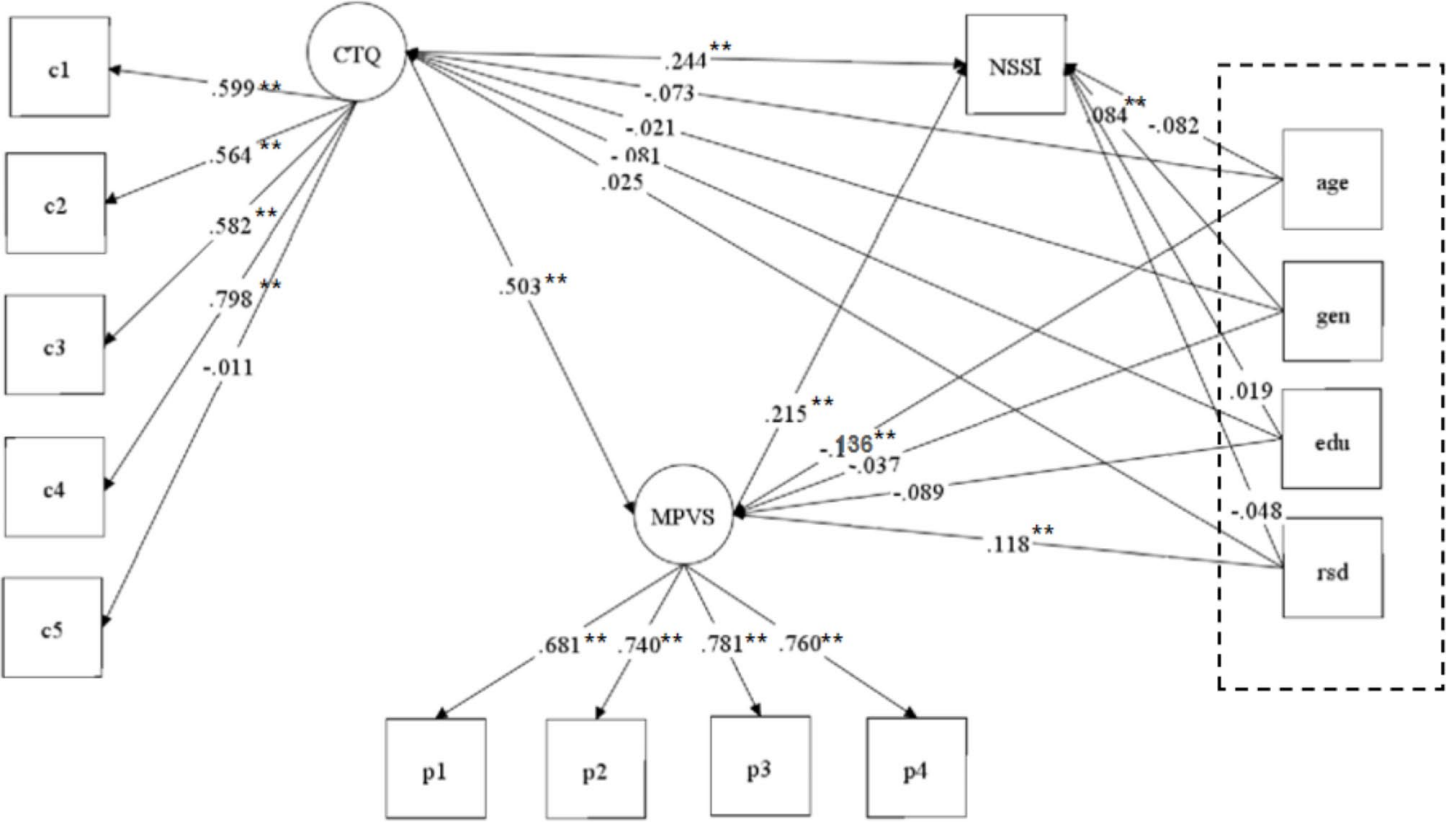
Mediation effect of peer victimization on the link between childhood trauma and non-suicide self-injury. Note: Variables in the dashed box are covariates, included: age, age; gen, gender; edu, education; rsd, resident. CTQ, Childhood Trauma Questionnaire; c1, emotional abuse; c2, physical abuse; c3, sexual abuse; c4, emotional neglect; c5, physical neglect; MPVS, Multidimensional peer victimization Scale; p1, physical victimization; p2, verbal victimization; p3, social manipulation; p4, attacks on property

Latent variable SEM was used to test the mediating role of peer victimization in the relationship between childhood trauma and NSSI (Fig. 2). The hypothetical model fit the data well (n = 1,783, CFI = 0.848, TLI = 0.801, RMSEA = 0.086, X2/df = 14.129). The standardized path coefficients from childhood trauma to peer victimization (β = 0.503, p < 0.001) and from peer victimization to NSSI (β = 0.215, p < 0.001) were statistically significant. Moreover, the direct effect of childhood trauma on NSSI was significant (β = 0.244, p < 0.001). The total effect in this model was 0.353, and the mediating effect was 0.109, which accounted for 30.9% of the total effect. The detailed effect sizes of all pathways in the model are presented in Table 2 It can be seen that peer victimization mediated the relationship between childhood trauma and NSSI and that childhood trauma still directly affected NSSI. Adolescents with traumatic experiences in childhood are more likely to experience greater peer victimization in adolescence, which may lead to increased NSSI. Besides, age has significant influences on MPVS, gender has significant influences on NSSI, residence has significant influences on MPVS.

Besides, due to unbalanced ratio of gender and resident in the study, male subjects and rural subjects were separately selected to make further SEM analysis, the results were similar to the total results. However, the influence of covariates on the main variables is different from the overall research results. Residence has significant influences on MPVS and CTQ in the males subjects, and gender has significant influences on NSSI in rural subjects. This part of results is presented in the appendix.

## Discussion

NSSI is a common and serious mental health problem among adolescents. With an increasing body of literature investigating the period of adolescence [49], the impact of early childhood experiences and peer relationship on adolescent problem behaviors is gaining more attention [50]. While several studies have explored the effects of childhood trauma and peer victimization on NSSI [51], the underlying relationships remain unclear. Thus, this study constructed a potential structural model to investigate how childhood trauma and peer victimization influence NSSI among Chinese adolescents. The results revealed that childhood trauma directly affected peer victimization as well as NSSI. Besides, peer victimization directly affected NSSI. And childhood trauma indirectly affected NSSI among Chinese adolescents through peer victimization. These results provide a basis for further exploration of the mechanism underlying NSSI in adolescents.

These findings are consistent with prior research indicating that trauma has a significant independent effect on NSSI [52]. The linear regression analysis shows that in childhood trauma, emotional abuse, physical abuse, emotional neglect and physical neglect both had significant effect on peer victimization. Consistent with previous findings, experiences of childhood emotional abuse and neglect can build maladaptive schemas for children, leading to the increase of the incidence of peer victimization [53]. Besides, people who were frequently victimized (including emotional abuse, physical abuse, etc. ) during childhood, they repeat and perpetuate an “aggressor-victim” interaction in their subsequent relationships [54]. In addition, some study thought that sexual abuse also play a important role in the relationship of early adolescent [55], which need to be estimated in future studies. Furthermore, physical abuse and emotional neglect had more effect on NSSI. The mental and psychological problems associated with physical abuse and neglect are varied. Research studies have found that physical abuse and neglect is associated with a large number of interpersonal, cognitive, emotional, behavioral, and substance abuse problems and psychiatric disorders [56]. In addition, Research shows physical abuse and emotional neglect had the strongest association with adult psychiatric disorders [57]. Therefore, the impact of physical abuse and emotional neglect should be given special attention during the growth of adolescents.

Different dimensions of childhood trauma may have different effects on NSSI. Child maltreatment [58], including emotional abuse [59], physical abuse, sexual abuse, physical neglect, and emotional neglect [60], have been confirmed to be significantly associated with NSSI [61], among which, physical and sexual abuse are more likely to increase the risk of suicide attempts [62]. In another study, emotional neglect and emotional abuse were identified as key factors in NSSI [63]. Childhood abuse affects the normal psychological development of adolescents and has been found to be associated with both depressive and NSSI behavior in adolescence [20]. Another study found that traumatic childhood experiences can lead to psychological dysfunction, which can trigger NSSI [64]. Several studies have also confirmed that a higher burden of adverse childhood experiences is associated with a higher risk of many adverse health, behavioral, psychological, and social outcomes [65]. Traumatic experiences in childhood not only have profound significance for individual psychological well-being and life quality, but also for the development of cognitive function. Several neurobiological studies have confirmed that individual exposure to childhood trauma may lead to a weakened hypothalamic-adrenal axis response [66], and NSSI has a moderating effect on the relationship between childhood trauma and hypothalamic-pituitary-adrenal axis function [67]. In summary, childhood trauma can cause psychological damage to individuals and can affect the structure and function of the brain, leading to long-lasting effects.

In the current study, the latent variable mediation model indicated that childhood trauma can further affect the NSSI of adolescents by influencing their experience of peer victimization. Similarly, our model has also been verified in the sample of individual male and rural subjects. In fact, several scholars have demonstrated that poor childhood experiences (bullying [68], Sexual victimization [69], Guardian incapacity [70], etc.) are among the strongest predictors of peer victimization outside the home during adolescence and later in adulthood [71]. The first potential explanation for this finding is that children who have experienced childhood trauma will repeat these patterns of interaction from their past family environment in their future interpersonal interactions [72]. D. W. Winnicott, a leading psychologist, argued that the formation and development of interpersonal relationships are largely affected by the relationship between the child and their main caregivers in early childhood [73]. Several recent studies have also reported that childhood trauma (maltreatment) in the family context is associated with later peer victimization [74]. Adolescents who have experienced childhood trauma may generalize the non-adaptive behaviors they learned from childhood to their extra-familial interactions, resulting in them being regarded as targets for bullying by peer groups [75]. It is worth noting that some children who have suffered childhood abuse are not only more likely to be bullied, but also to become bullies. Studies have found that these children exhibit higher levels of aggression and antisocial behavior [76], which makes them more likely to hurt others or themselves. Another explanation is that childhood trauma can affect adolescents’ perceptions of interpersonal relationships, resulting in inappropriate behavioral responses. The pioneer of object relation theory, Melanie Klein, found that childhood attachment to family not only affects post-adult relationships, but also alters the way an individual feels and reacts when in the presence of negativity [77]. Disorganized-disoriented parent-child attachment patterns shape an insecure avoidant attachment style; an individual with this attachment pattern tends to exhibit characteristics of jealousy, aggression, and sensitivity to negative stimuli in the process of interpersonal communications, which makes them feel unfulfilled and hurt in the relationship [78]. Certain characteristics, like indecisiveness, sensitivity, unconditional compliance, and so on can make a victimized children more likely to be the target of other forms of assault [75].

Peer victimization is a significant predictor of NSSI [79]. In all dimensions of peer victimization, physical victimization, verbal victimization and attacks on property had more effect on NSSI under controlling other related factors. Similar results have been found in previous studies. In a cross-lagged panel study, sleep problems may be a consequence of physical victimization [80]. And sleep problems is linked with NSSI, including NSSI history and intensity of urges to engage in NSSI [81]. Moreover, a study of juvenile offenders found that direct, indirect physical assault and verbal assault are common injuries to juvenile offenders [82]. The various forms of victimization experienced by these juvenile offenders affect their physical and psychological well-being and lead to various problematic behaviors. Although there are few studies on attacks on property, but it is also an important factor in victimization. It is pointed out in some researches that peer victimization is the most salient peer stressor to affect physical health outcomes in adolescence [83], could induce more NSSI [27].

In the general stress theory of Agnew, personal pressure or extra-personal stressors can lead to negative emotions and behaviors [84], and some teenagers could adopt dangerous behaviors such as NSSI in order to release these bad feelings [85]. Moreover, peer victimization is a major source of stress [84], and adolescents who have been bullied often have difficulty adjusting to school or society, which may increase the risk of them engaging in self-hurt behaviors. The interpersonal model proposes that NSSI can be used by teenagers as a negative coping strategy to reduce stress or tension caused by adverse interpersonal events [41]. However, as NSSI increases, peer bullying may be more prevalent and can lead to more subsequent NSSI [86].

Childhood trauma and peer victimization can be seen as injuries from the outside while NSSI, by definition, is a form of self-inflicted injury [87]. Actions play a key role in providing ‘meaning’ for the feelings [88]; in other words, self-injury behavior reflects, to some extent, the individual’s inner feelings after experiencing external abuse and harm. Hence, an understanding of the interaction between these three types of injury may have important implications for further understanding of the true psychological state of adolescents with NSSI, and thus, appropriate psychological interventions. Although there is still much room for further exploration of the internal mechanisms underlying the connections between the three concepts, several available studies offer potential explanations for the relationships observed in the current study. For example, several recent studies have demonstrated that adolescents who are exposed to negative parents may learn that they are inferior, have vulnerable self-esteem, and may become less able to express their true feelings [89]. Coupled with the experience of peer victimization, this can make it difficult for such adolescents to obtain sufficient social support for their psychological issues, resulting in the adoption of negative coping behaviors, such as NSSI, to express their anger [90]. Studies also indicate that both peer victimization [91]. and childhood trauma [92] are associated with negative long-term and short-term psychiatric, educational, and medical outcomes and increased risk of self-injurious behavior. In conclusion, childhood trauma and peer victimization are among the antecedent factors that contribute to the development of NSSI. Thus, it is important that both researchers and clinicians pay attention to the impact of the early environment and current circumstances on the mental health of adolescents. Timely interventions will be needed to shift the trajectory of development in a positive direction, providing beneficial effects on the physical and mental health of adolescents.

In the study, we also found that gender had a significant effect on NSSI. The finding is consistent with past researches, females report a higher prevalence of NSSI compared to males [93], in addition to literature reporting that NSSI behaviors are more common in females aged 16–19 years [94]. There is no gender difference in childhood trauma, which is different from previous studies. Some researches showed that women experienced more childhood trauma, especially with emotional abuse [95], compared to men. Possibly because of the development of the economy, the concept of “son preference” of out-dated ideas is changed by “gender equality”, girls also get better care [96]. And there was no significant gender difference in peer victimization. The similar results were found in previous study, and depressive symptoms and stressful life events mediate the association between peer victimization and NSSI in adolescents, [97]. Previous studies have noted significant gender differences in the effects of childhood trauma on peer victimization. A prospective study reported that females who suffered child sexual abuse were at increased risk of peer victimization compared to males [98]. However, some studies also found that there are no gender differences in physical violence in childhood trauma, i.e., both boys and girls who are physically abused in childhood are at increased risk of peer intimidation and physical assault in adolescence [99].This could be explored further in future studies.

Finally, there are several limitations of this study that should be noted. First, the demographic distribution of the same was uneven; the proportion of female subjects was much larger than that of male subjects, and the proportion of urban subjects was much larger than that of rural subjects. This may have had an impact on the findings and the effects of the covariates. Second, there is a long period of time between childhood and young adulthood, and during this time there are likely many other influences on the development of NSSI that have not been explored in this study. Third, given the samples were collected from the clinical, most of subjects are patients with emotional disorders, thus patients with depression are not completely excluded. Fourth, other demographic variables like family income were not collected in this study. We will consider to include more demographic variable in future study. Fifth, there are memory bias existed to impact the assessment of childhood trauma, and memory bias will also be affected by gender, age, residence, education level, etc. In future research, we can consider using longitudinal research or combining self-evaluation with other evaluation methods to reduce recall bias to improve the validity of the study. Nonetheless, this study provides an evidence base for further investigation of the risk factors for NSSI. In the future, longitudinal studies should explore the causal mechanisms underlying the influence of childhood trauma on NSSI and the potential roles of other important risk factors.

## Conclusion

In conclusion, this study used SEM to model the relationship between the latent variables of childhood trauma (c1, emotional abuse; c2, physical abuse; c3, sexual abuse; c4, emotional neglect; c5, physical neglect) and peer victimization (p1, physical victimization; p2, verbal victimization; p3, social manipulation; p4, attacks on property). The SEM analysis demonstrated that peer victimization mediates the relationship between childhood trauma and NSSI. In addition, several covariates (such as age, gender, education level, and place of residence) regulated the relationship between peer victimization and NSSI. These results suggest that researchers and clinicians should pay attention to the associations between childhood trauma, peer victimization, and NSSI, as well as the influences of age, gender, education level, and place of residence on adolescent problem behaviors. This may help to inform the development of interventions for adolescent psychological and behavioral problems.

## Electronic supplementary material

Below is the link to the electronic supplementary material.

Supplementary Material 1

Supplementary Material 2

Supplementary Material 3

## Data Availability

The datasets generated and/or analyzed during the current study are not publicly available due to limitations of ethical approval involving the patient data and anonymity but are available from the corresponding author on reasonable request.
