# Supplementary material for: Childhood trauma, peer victimization, and non-suicidal self-injury among Chinese adolescents: a latent variable mediation analysis
Source: BMC Psychiatry. 2023 Jun 15;23:436. doi: 10.1186/s12888-023-04848-z (PMC10268482; doi:10.1186/s12888-023-04848-z)
Supplement: Supplementary file 2 — Supplementary Material 2 [file 12888_2023_4848_MOESM2_ESM.docx]

**Additional file 2**

**Supplementary Table S1.Structural model assessment**

|  | Path | Estimate | S.E. | Est. / S.E. | Standardized 95% CI  Low High | | P-value |
| --- | --- | --- | --- | --- | --- | --- | --- |
| Direct Effect | CTQ→NSSI | 0.244 | 0.032 | 7.611 | 0.181 | 0.327 | ＜0.001 |
| Indirect Effects | CTQ→MPVS→NSSI | 0.109 | 0.017 | 6.265 | 0.075 | 0.154 | ＜0.001 |
| Total Effect | | 0.352 | 0.026 | 13.544 | 0.302 | 0.404 | ＜0.001 |
| Indirect effect  (%, total indirect effect/total effect) | | 0.109  (30.9%) |  |  |  |  |  |

**Note:** CTQ, Childhood Trauma Questionnaire; MPVS, Multidimensional peer victimization Scale; NSSI, non-suicide self-injury.

**Supplementary Table S2 the effect of Covariates in the Structural model**

|  | Path | StdYX  Estimate | Standardized 95% CI  Low High | | P-value |
| --- | --- | --- | --- | --- | --- |
| Covariates | Age→CTQ | -0.073 | -0.315 | 0.057 | ＞0.05 |
|  | Gen→CTQ | -0.032 | -0.592 | 0.236 | ＞0.05 |
|  | Edu→CTQ | -0.081 | -0.306 | 0.042 | ＞0.05 |
|  | Rsd→CTQ | 0.025 | -0.125 | 0.553 | ＞0.05 |
|  | **Age→MPVS** | **-0.136** | **-0.241** | **-0.046** | **＜0.01** |
|  | Gen→MPVS | -0.037 | -0.423 | 0.041 | ＞0.05 |
|  | **Edu→MPVS** | **-0.089** | **-0.194** | **-0.003** | **＜0.01** |
|  | **Rsd→MPVS** | **0.118** | **0.229** | **0.514** | **＜0.01** |
|  | Age→NSSI | -0.082 | -0.954 | 0.027 | ＞0.05 |
|  | **Gen→NSSI** | **0.084** | **1.048** | **3.116** | **＜0.01** |
|  | Edu→NSSI | 0.019 | -0.329 | 0.514 | ＞0.05 |
|  | Rsd→NSSI | -0.048 | -1.368 | 0.224 | ＞0.05 |

**Note:** Age, age; Gen, gender; Edu, education; Rsd, resident; CTQ, Childhood Trauma Questionnaire; MPVS, Multidimensional peer victimization Scale; NSSI, non-suicide self-injury.
