# Supplementary material for: Childhood trauma, peer victimization, and non-suicidal self-injury among Chinese adolescents: a latent variable mediation analysis
Source: BMC Psychiatry. 2023 Jun 15;23:436. doi: 10.1186/s12888-023-04848-z (PMC10268482; doi:10.1186/s12888-023-04848-z)
Supplement: Supplementary file 3 — Supplementary Material 3 [file 12888_2023_4848_MOESM3_ESM.docx]

**Additional file 3**

**Figure S1.** Mediation effect of peer victimization on the link between childhood trauma and non-suicide self-injury in male subjects.

**
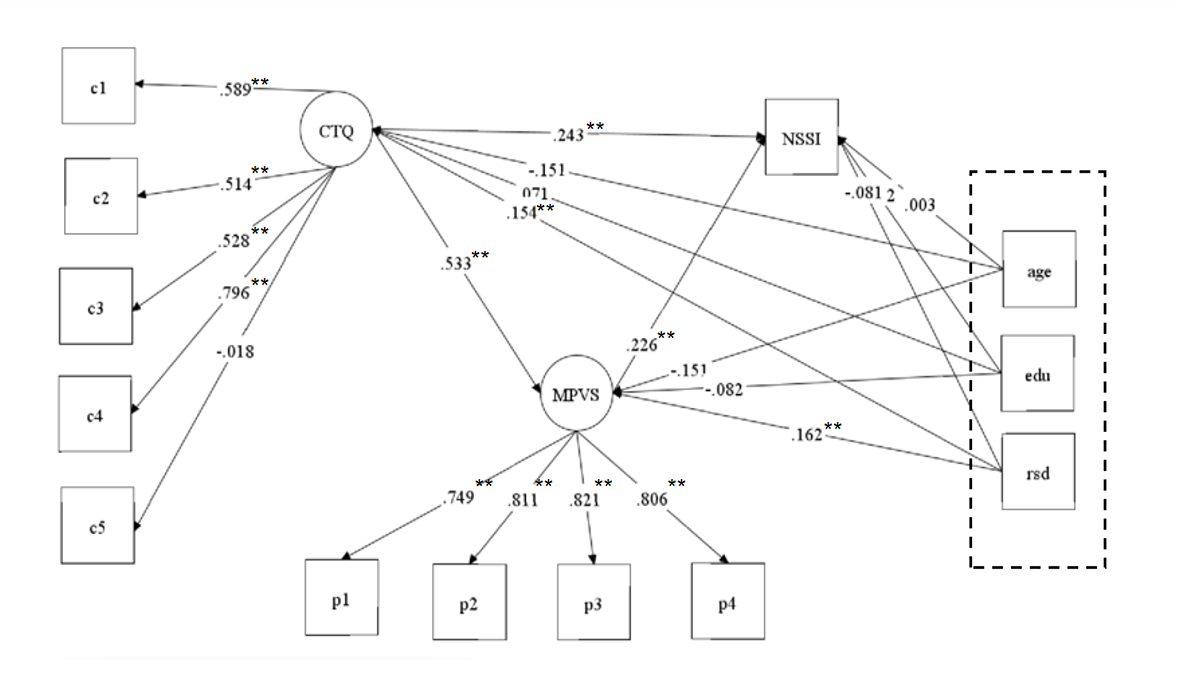
Note：**The above is the whole mediation model and the below is the significant path presentation. Variables in the dashed box are covariates, included: age, age; edu, education; rsd, resident.

CTQ, Childhood Trauma Questionnaire; c1, emotional abuse; c2, physical abuse; c3, sexual abuse; c4, emotional neglect; c5, physical neglect; MPVS, Multidimensional peer victimization Scale; p1, physical victimization; p2, verbal victimization; p3, social manipulation; p4, attacks on property; NSSI, non-suicide self-injury.

**Figure S2.** Mediation effect of peer victimization on the link between childhood trauma and non-suicide self-injury in rural subjects.


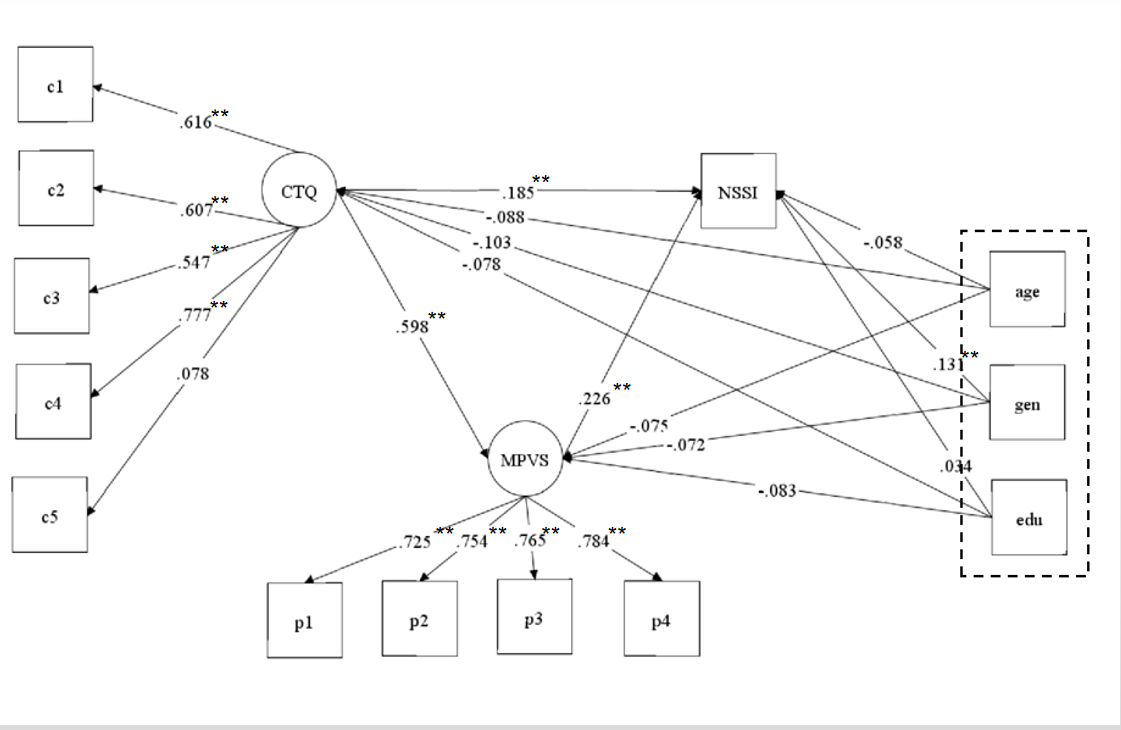


**Note:** The above is the whole mediation model and the below is the significant path presentation. Variables in the dashed box are covariates, included: age, age; gen, gender; edu, education.

CTQ, Childhood Trauma Questionnaire; c1, emotional abuse; c2, physical abuse; c3, sexual abuse; c4, emotional neglect; c5, physical neglect; MPVS, Multidimensional peer victimization Scale; p1, physical victimization; p2, verbal victimization; p3, social manipulation; p4, attacks on property; NSSI, non-suicide self-injury.
